# Prognostic accuracy of head computed tomography for prediction of functional outcome after out-of-hospital cardiac arrest: Rationale and design of the prospective TTM2-CT-substudy

**DOI:** 10.1016/j.resplu.2022.100316

**Published:** 2022-10-12

**Authors:** Margareta Lang, Christoph Leithner, Michael Scheel, Martin Kenda, Tobias Cronberg, Joachim During, Christian Rylander, Martin Annborn, Josef Dankiewicz, Nicolas Deye, Thomas Halliday, Jean-Baptiste Lascarrou, Thomas Matthew, Peter McGuigan, Matt Morgan, Matthew Thomas, Susann Ullén, Johan Undén, Niklas Nielsen, Marion Moseby-Knappe

**Affiliations:** a Department of Clinical Sciences Lund, Radiology, Lund University, Helsingborg Hospital, Helsingborg, Sweden; b Department of Neurology and Experimental Neurology, Charité-Universitätsmedizin Berlin, Germany; c Department of Neuroradiology, Charité-Universitätsmedizin Berlin, Germany; d Berlin Institute of Health at Charité, Universitätsmedizin Berlin, Germany; e Department of Clinical Sciences Lund, Neurology, Lund University, Skåne University Hospital, Lund, Sweden; f Department of Clinical Sciences Lund, Anaesthesia and Intensive Care, Lund University, Skåne University Hospital, Malmö, Sweden; g Department of Surgical Sciences, Anaesthesia and Intensive Care, Uppsala University, Uppsala, Sweden; h Department of Clinical Sciences Lund, Anesthesia & Intensive Care, Lund University, Helsingborg Hospital, Helsingborg, Sweden; i Department of Clinical Sciences Lund, Cardiology, Lund University, Skåne University, Lund, Sweden; j Department of Medical and Toxicological Intensive Care Unit, Lariboisière Hospital, Paris, France; k Department of Operation and Intensive Care, Linköping University Hospital, Linköping, Sweden; l Médecine Intensive Réanimation, University Hospital Center, Nantes, France; m Intensive Care Unit, University Hospitals, Bristol and Weston, England, United Kingdom; n Regional Intensive Care Unit, Royal Victoria Hospital, Belfast, Northern Ireland, United Kingdom; o Department of Intensive Care, the Royal Perth Hospital, Perth, Australia; p Department of Intensive Care, The University Hospital of Wales, Cardiff, United Kingdom; q School of Medicine, Curtin University, Perth, Australia; r University Hospitals, Bristol and Weston, United Kingdom; s Clinical Studies Sweden – Forum South, Skåne University Hospital, Lund, Sweden; t Department of Clinical Science Lund, Lund, Sweden; u Department of Operation and Intensive Care, Hallands Hospital Halmstad, Halmstad, Sweden

**Keywords:** Neuroprognostication, Computed tomography, Hypoxic-ischaemic encephalopathy (HIE), Cardiac arrest, Targeted temperature management, Outcome, GWR grey-white matter ratio

## Abstract

**Background:**

Head computed tomography (CT) is a guideline recommended method to predict functional outcome after cardiac arrest (CA), but standardized criteria for evaluation are lacking. To date, no prospective trial has systematically validated methods for diagnosing hypoxic-ischaemic encephalopathy (HIE) on CT after CA. We present a protocol for validation of pre-specified radiological criteria for assessment of HIE on CT for neuroprognostication after CA.

**Methods/design:**

This is a prospective observational international multicentre substudy of the Targeted Hypothermia versus Targeted Normothermia after out-of-hospital cardiac arrest (TTM2) trial. Patients still unconscious 48 hours post-arrest at 13 participating hospitals were routinely examined with CT. Original images will be evaluated by examiners blinded to clinical data using a standardized protocol. Qualitative assessment will include evaluation of absence/presence of “severe HIE”. Radiodensities will be quantified in pre-specified regions of interest for calculation of grey-white matter ratios (GWR) at the basal ganglia level. Functional outcome will be dichotomized into good (modified Rankin Scale 0–3) and poor (modified Rankin Scale 4–6) at six months post-arrest. Prognostic accuracies for good and poor outcome will be presented as sensitivities and specificities with 95% confidence intervals (using pre-specified cut-offs for quantitative analysis), descriptive statistics (Area Under the Receiver Operating Characteristics Curve), inter- and intra-rater reliabilities according to STARD guidelines.

**Conclusions:**

The results from this prospective trial will validate a standardized approach to radiological evaluations of HIE on CT for prediction of functional outcome in comatose CA patients.

The TTM2 trial and the TTM2 CT substudy are registered at ClinicalTrials.gov NCT02908308 and NCT03913065.

## Background

Post-cardiac arrest brain injury is the result of a transient ischaemia with subsequent reperfusion which may manifest as hypoxic-ischaemic encephalopathy (HIE) on neuroimaging.^1^ Guidelines recommend that the presence of “diffuse and extensive anoxic injury” on head computed tomography (CT) or magnetic resonance imaging (MRI) may be used as a predictor of poor neurological outcome after cardiac arrest (CA), yet the level of evidence is low.^2^ Despite its widespread clinical use, meta-analyses have concluded that the majority of prognostic studies on neuroimaging are single centre studies limited by a retrospective design.^2^, ^3^, ^4^, ^5^ Furthermore, radiological evaluation of HIE on CT for prognostic purposes lacks standardized criteria.^2^ Its performance for outcome prediction in clinical practice is likely influenced by both interrater variability (at least partly due to lack of standardization of the CT evaluation process) and technical parameters – inter-scanner variability.^6^, ^7^, ^8^, ^9^, ^10^, ^11^ This potentially introduces a risk for a false pessimistic prediction of poor outcome.^7^, ^8^

To improve prognostic performance, various approaches to quantitative CT analysis have been investigated, but their clinical application is limited by the lack of consensus on which regions of interest (ROI) and cutoff values are most valid.^4^, ^12^, ^13^, ^14^ We previously performed studies to compare prognostic accuracies and interrater variabilities of various qualitative, semi-quantitative and quantitative methods on CT for prediction of poor functional outcome.^13^, ^15^ We have used these results to establish Standard Operating Procedures (SOP) for both qualitative and quantitative radiological evaluations of head CT images in CA patients. Here we present these SOP and the protocol for their validation in a prospective international observational trial, the CT substudy of the targeted Hypothermia versus targeted Normothermia after out-of-hospital cardiac arrest (TTM2) trial.^16^

*Hypotheses of the TTM2 CT substudy are:*

In patients unconscious more than 48 h after CA:

1. A standardized qualitative assessment of head CT has a higher prognostic accuracy than non-standardized qualitative assessment (current reference standard) for poor outcome prediction
2. The following findings are predictive of a poor functional outcome with 0% false positive ratio (FPR):
  a) Definite signs of “severe HIE” diagnosed using our SOP for qualitative assessment.
  b) Grey-white matter ratio (GWR) at the basal ganglia level below pre-specified cutoffs (1.10 and 1.15) determined using our SOP for quantitative assessment.
  c) Automated, atlas-based GWR on the basal ganglia level (auto GWR) below 1.10 as described by Kenda *et al.*^17^.

3. The prognostic accuracies of the CT analysis methods described above is independent from the type of CT scanner, previous use of CT contrast agent and targeted temperature management.
4. No patient with “severe HIE” on head CT will have low blood levels of the brain injury marker Neurofilament light (NFL) (0% FPR).^18^
5 The reliability of agreement for prediction of poor outcome using qualitative and quantitative assessment of head CT will be good (Fleisś kappa > 0.7).^19^

## Methods/design

### Participants and ethical consent

The TTM2-trial (Clinicaltrials.gov NCT02908308) was an international, multicentre, parallel group, investigator-initiated trial which randomised 1900 adult patients with an out-of-hospital cardiac arrest to a target temperature of 33°C or to a strategy to maintain normothermia and early treatment of fever (≥37.8°C).^16^, ^20^

**Fig. 1:**
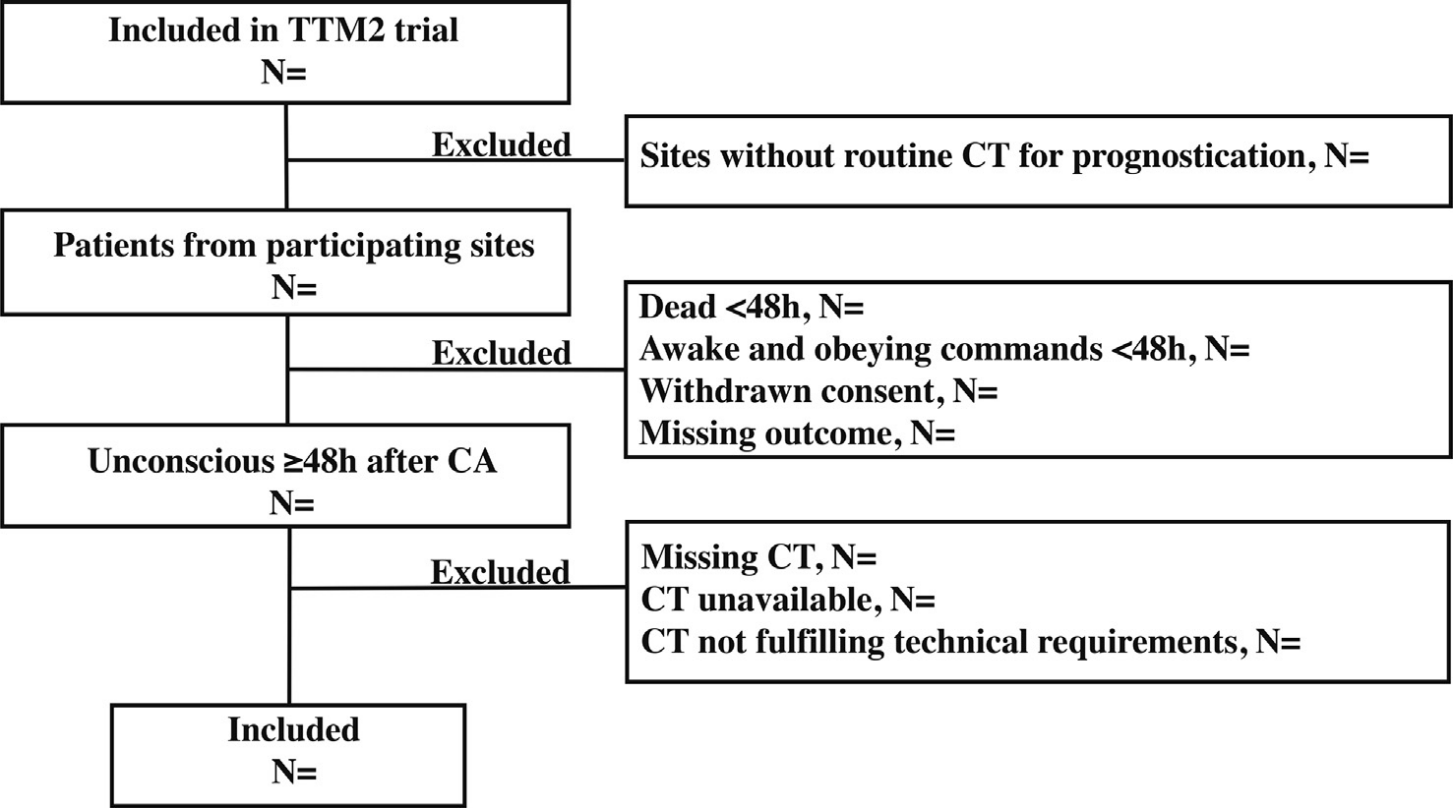
**Flowchart patient inclusion for CT substudy** CT, head computed tomography; N, number of patients; h, hours.

The TTM2-CT-substudy (Clinicaltrials.gov NCT039130659) is a prospective international multicentre observational study examining the prognostic accuracy of head CT for prediction of functional outcome after CA. Between 11/2017 and 01/2020, patients were recruited at 13 TTM2 sites routinely examining patients still unconscious at 48 hours post-arrest with head CT (Flowchart Fig. 1). Unconsciousness was defined as not obeying verbal commands and a response to painful stimulus < 4 on the Full-Outline of Unresponsiveness (FOUR) motor response (at best localizing pain).^20^, ^21^.

Each participating country obtained approval by the appropriate ethics committee prior to patient enrolment.^16^ Patients were treated according to the TTM2-trial protocols regarding inclusion and exclusion criteria, randomisation, clinical management, neurological prognostication, decisions on withdrawal of life-sustaining therapy (WLST) and follow-up.^20^, ^22^, ^23^

### Procedure

CT images were initially evaluated by radiologists at the patient’s local hospital without any pre-specified criteria for evaluation and results were available to the treating team when predicting outcome. The results of the local radiologists’ evaluations were entered into the electronic case report form (eCRF) as a “yes”/”no” answer to the question “Are there signs of global, diffuse or bilateral multifocal ischaemic injury on CT?”. The date and time for examination and the number of CT examinations performed was also reported.

### Technical requirements and data collection

For this substudy, collection of the original Digital Imaging and Communications in Medicine (DICOM) images was initiated after randomisation of the last patient in the TTM2 trial. Images were collected and stored in a digital database labelled with the patient́s trial identification number. Data will be kept according to national legislation for record keeping.

All types of scanners and software were permitted, and specifics for each scan will be registered and analysed. Technical prerequisites for including CTs in the analysis are: Axial slices of 4–5 mm slice thickness of the entire brain available with tube voltage 120 kV. We will extract the following technical metadata: time of CT examination, manufacturer, scanner and convolution kernel. Radiological evaluations will be started after this study protocol has been accepted for publication. Examiners will either be radiologists or neurologists, all with clinical experience in CT evaluation of CA patients.

### Outcomes

Patient outcomes will be poor functional outcome six months after randomisation, defined as modified Rankin Scale (mRS) 4 -6.^22^ Serum levels of neurofilament light (NFL) at 48 h post-randomisation analysed with an Elecsys® electrochemiluminescence immunoassay (ECLIA) will be used as a surrogate marker of brain injury.^24^ The level of agreement between examiners evaluations will be reported using measures of inter- and intra-rater variability as described below.

### Standardized operating procedure for CT evaluation

#### SOP qualitative analysis

**Fig. 2:**
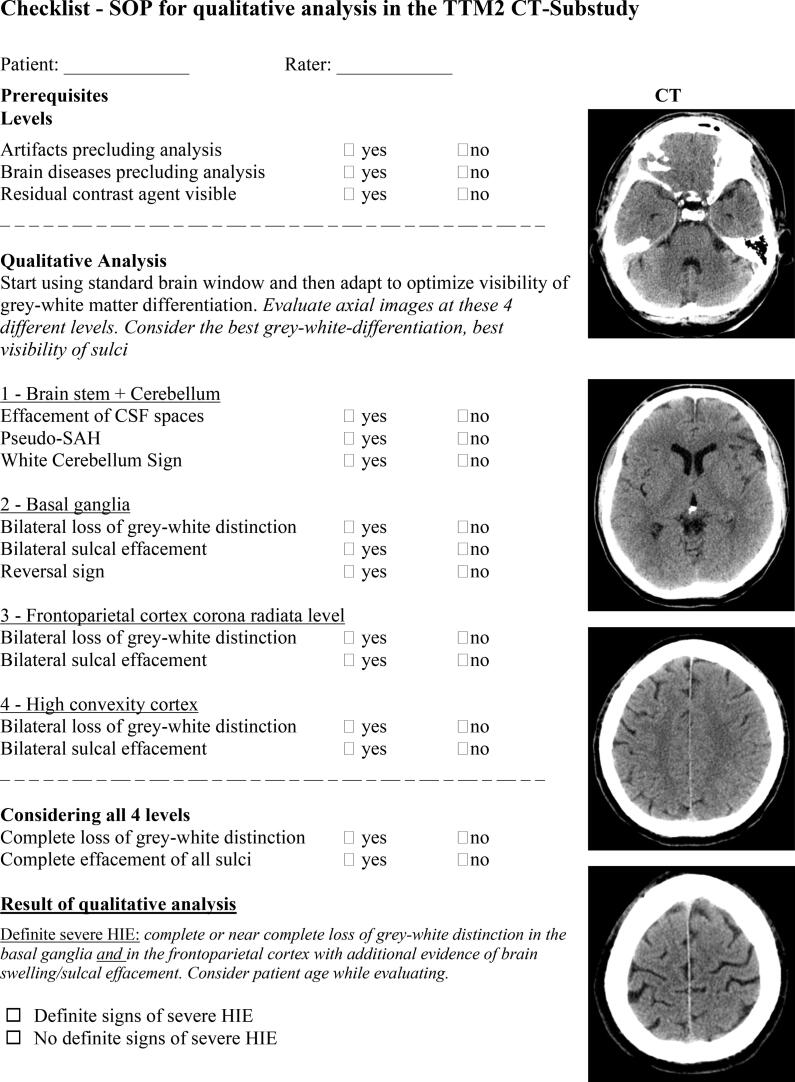
**SOP checklist for qualitative analysis** Standardised operating procedure checklist for qualitative radiological evaluation. CSF; cerebrospinal fluid, SAH; subarachnoid haemorrhage, HIE; hypoxic-ischaemic encephalopathy.

Qualitative analysis will be performed according to the checklist in Fig. 2. The full SOP can be seen in S1. The examiners will first determine whether the prerequisites for qualitative analysis are fulfilled; that there are no imaging artifacts precluding analysis, and absence of significant intracranial pathologies such as haemorrhage, stroke, tumour, extensive calcification which could interfere with analysis. Importantly, CTs with moderate brain atrophy, moderate vascular leukoencephalopathy or chronic strokes not affecting analysis of grey-white matter distinction or sulcal effacement at the basal ganglia level and bilateral frontoparietal regions should be used for analysis. The examiner will also report whether residual contrast agent from for example coronary angiography is visible.

Image evaluation starts by applying a standard “brain window” (WW: 80, WL: 40) which is then adapted to optimize visibility of grey-white-matter differentiation.^25^ The focus of evaluation will be on distinction of grey and white matter and/or on sulcal effacement evaluated at the following levels: 1) brainstem + cerebellum, 2) basal ganglia, 3) cortex at corona radiata level, 4) high convexity cortex. Cerebrospinal fluid spaces will be evaluated considering the age of the patient (i.e physiologically high brain volume and small subarachnoid spaces in young patients).

Additional characteristics of severe HIE may include “Pseudo-subarachnoid haemorrhage sign” (hyperdense cerebrospinal fluid signal in basal cisterns or over the convexity), “reversal sign” (lower radiodensity of grey compared to white matter structures) and “white cerebellum sign” (higher radiodensity of cerebellum as compared to cerebral hemispheres).^26^, ^27^

The main question to be answered after evaluation of the entire CT is “Are there definite signs of severe HIE?” This question should be answered with “yes”, if complete or near-complete loss of grey-white distinction is noted both in the 1) basal ganglia and 2) in the frontoparietal lobes bilaterally with additional evidence of brain swelling/sulcal effacement. Severe HIE will also be diagnosed from complete sulcal effacement when residual grey-white distinction is present.

#### SOP quantitative analysis – grey-white matter ratio

**Fig. 3:**
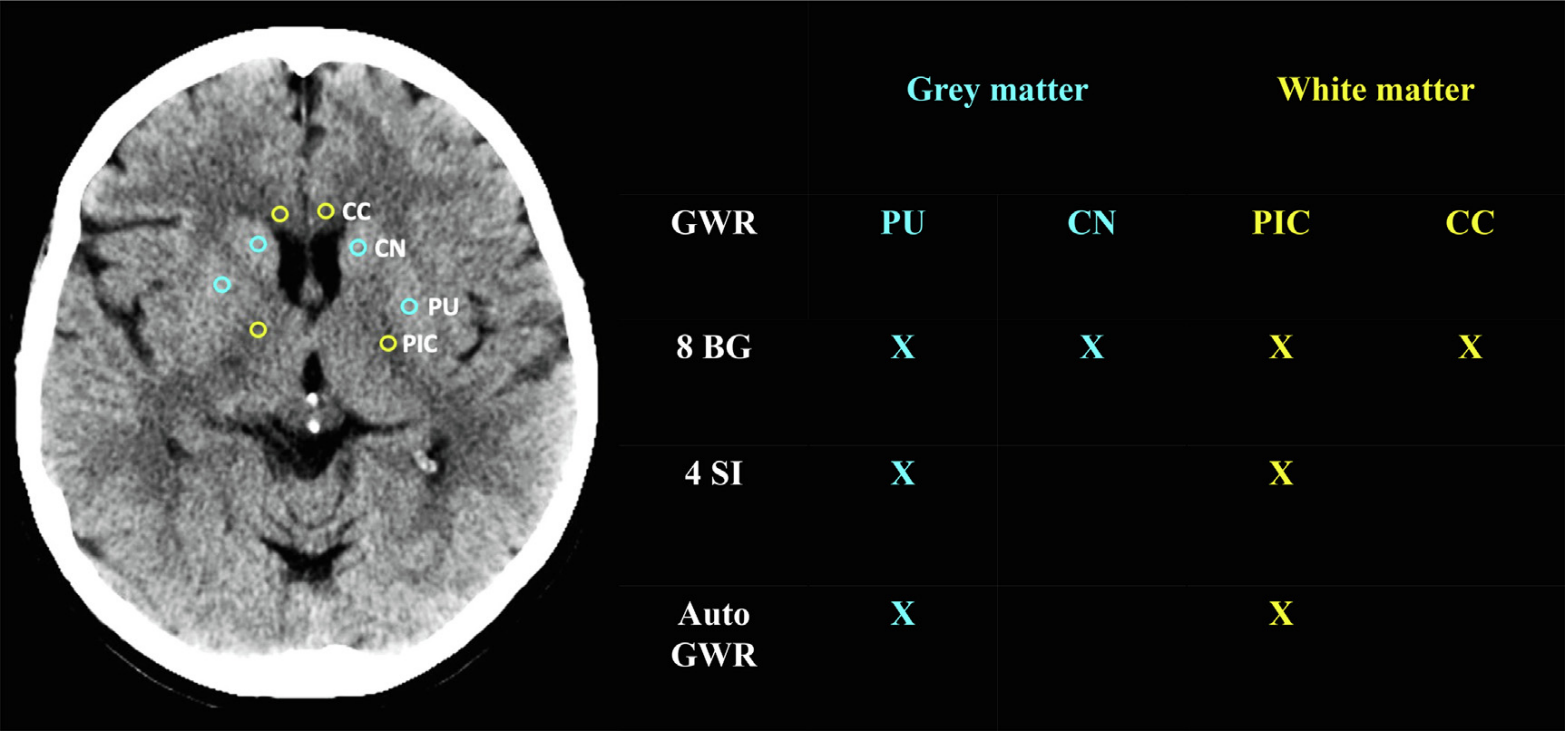
**SOP for the qualitative measurement of grey-white matter ratio** Placement of Regions of Interest (ROI) for determination of the grey-white matter ratio (GWR) at the basal ganglia level including 8 or 4 ROIs.^12^, ^13^ Yellow indicates white matter ROIs and blue indicates ROIs in the grey matter. All axial slices containing basal ganglia structures should be evaluated and ROIs placed bilaterally in the slice best representative of that target region. Thus, these 8 ROIs may be placed in different slices: 1) Putamen (PU); 2) Head of the caudate nucleus (CN); 3) Posterior limb of the internal capsule (PIC), and 4) Genu of the corpus callosum (CC). In case of complete loss of grey-white distinction, use ventricles and midline as landmarks. In some patients with severe HIE radiodensity is similar in grey and white matter, exact location of target regions cannot always be determined. Nonetheless, ROIs should be placed and patients should not be excluded from GWR determination. Crosses indicate the ROIs included in each grey-white matter ratio method: 8 BG (basal ganglia model), 4SI (simple model) and auto GWR (automated GWR determination).^17.^

For quantitative analysis, circular ROIs (0.1 cm^2^) will be placed manually in pre-defined anatomical regions of grey and white matter bilaterally at the basal ganglia level (Figs. 3, S2). The examiners are instructed to check the Hounsfield Units (HU) during measurements to ensure ROI placement in an area where the radiodensity is representative of the entire target brain region. Positioning ROIs in focal hypo- or hyperdensities, e.g. resulting from small vascular lesions, calcifications or noise must be avoided. Grey-white-matter ratios (GWR) will be calculated as the average density of the grey matter ROIs divided by the average density of the white matter ROIs using either 8 ROIs or for a simplified version, 4 ROIs at the basal ganglia level (Fig. 3).

#### Automated density measurements

In addition to our qualitative and quantitative SOPs, we will apply automated techniques for CT evaluation. First, we will validate an already published algorithm for automated GWR-assessment. Scans fulfilling technical prerequisites as stated above and without significant artifacts or acute/chronic pathologies other than HIE will be analysed. CT scans will be co-registered to an MRI-based digital standard brain using the free analysis software FSL-FMRIB Software v5.0 by Analysis Group, FMRIB, Oxford, UK) as previously described.^17^ Tissue probability maps derived from standard brain atlases will then be used to automatically identify anatomical regions on each CT scan. GWR will be calculated by the densities of the putamen and of the posterior limb of the internal capsule (Fig. 3).^17^

### Plan for statistical analysis

The reporting of results will follow the recommendations of the Standards for Reporting Diagnostic Studies (STARD).^28^ We will present a flow-chart of included and excluded patients, and reasons for exclusion (Fig. 1). We will describe patient data as displayed in Table 1.

**Table 1:** Example table for clinical characteristics and baseline variables.

	**Included** **N=**	**Excluded** **N=**
**Demographic characteristics**		
Age (years)	Mean (SD)	Mean (SD)
Male	N (%)	N (%)
**Time from CA to first CT performed**≥**48 h**	Median (IQR)	Median (IQR)
**Characteristics of cardiac arrest**		
Minutes from CA to ROSC	Median (IQR)	Median (IQR)
First monitored rhythm on ECG shockable	N (%)	N (%)
Bystander witnessed CA	N (%)	N (%)
Bystander CPR performed	N (%)	N (%)
Cardiac arrest at the place of residence	N (%)	N (%)
**Clinical characteristics on hospital admission**		
Corneal reflexes bilaterally absent on hospital admission	N (%)	N (%)
Pupillary reflexes bilaterally absent on hospital admission	N (%)	N (%)
ST-segment elevation myocardial infarction	N (%)	N (%)
Arterial lactate level on admission mmol/L	Mean (SD)	Mean (SD)
Circulatory shock on admission	N (%)	N (%)
Randomised to hypothermia	N (%)	N (%)
**Medical history**		
Hypertension	N (%)	N (%)
Dementia	N (%)	N (%)
Cerebrovascular disease	N (%)	N (%)
Hemiplegia	N (%)	N (%)
Diabetes	N (%)	N (%)
Myocardial infarction	N (%)	N (%)
Heart failure NYHA III or IV	N (%)	N (%)
Renal failure	N (%)	N (%)
Moderate or severe liver failure	N (%)	N (%)
Charlson comorbidity index	Median (IQR)	Median (IQR)
**Functional outcome after 180 days**		
Good outcome (mRS 0–3)	N (%)	N (%)
Poor outcome (mRS 4–6)	N (%)	N (%)
**Structured assessment of neurological outcome at 180 days**		
mRS 0	N (%)	N (%)
mRS 1	N (%)	N (%)
mRS 2	N (%)	N (%)
mRS 3	N (%)	N (%)
mRS 4	N (%)	N (%)
mRS 5	N (%)	N (%)
mRS 6	N (%)	N (%)
**Withdrawal of life-sustaining therapy (WLST)**		
WLST performed	N (%)	N (%)
WLST neurological reason only	N (%)	N (%)
WLST neurological based on CT	N (%)	N (%)

Data will be presented in numbers (percentages) for categorical variables, and in mean (standard deviation) or median (interquartile range) as appropriate for continuous variables.

#### Prognostic accuracies

For qualitative and quantitative evaluations, we will present sensitivities and specificities for prediction of poor functional outcome (mRS 4–6 at six months) with 95% confidence intervals (CI) calculated using Wilsońs method. Results will be reported separately for each examiner to avoid errors by averaging measurements by different investigators. Both for the manual and automated GWR evaluations we will validate cutoff values 1.10 and 1.15 for prediction of poor outcome. Several prior studies, including our own, suggest certainly poor outcome at GWR threshold below 1.10, yet at a cost of low sensitivity. To increase sensitivity we also validate the threshold 1.15.^3^, ^4^, ^15^

The overall prognostic performance for good versus poor functional outcome will also be tested by the area under the receiver-operating characteristics curve (AUROC) with 95% confidence intervals. P-values will be calculated based on a test of difference in AUROC using the method of DeLong.

#### Influence of targeted temperature management

Based on previous results, we do not expect that targeted temperature management influences the prognostic accuracies of CT.^29^ However, we will evaluate whether the approach to temperature management may have had any effect of the false positive predictions of poor outcome in our cohort.

#### Serum levels of neurofilament light as a surrogate marker of brain injury

Since results from CT evaluations by local radiologists were available upon clinical decision-making, we want to gather indirect evidence whether life-sustaining therapy withdrawn for neurological reasons (in this case based on CT findings) may have been leading to self-fulfilling prophecies. We will therefore investigate whether severe HIE was diagnosed from head CT in patients with low blood levels of neurofilament light, which indicate little or no brain injury.^18^

#### Reliability of agreement and intrarater variability

Fleisś kappa will be calculated as a measure of the reliability of agreement for qualitative analysis between investigators (local radiologists and study investigators) and between study investigators.^19^ With evaluation of n = 200 images, at least five blinded study examiners would yield a precision of 0.044 with 95% CI for Fleiss Kappa. To assess the intrarater variability (Coheńs Kappa), each examiner will re-examine 20% of images using the qualitative and quantitative SOP in evaluations performed independently from their first assessments.

#### Technical issues

A subgroup analysis will examine the group-wise difference in manually determined GWR between the different CT manufacturers, scanners, and convolution kernels.

### Exploratory analyses

Exploratory analyses will include prediction of outcome using quantification of regional brain water uptake, in patients with two or more CTs as well as other artificial intelligence-based methods.^30^

## Discussion

We present the study protocol of a prospective international multicentre trial which aims to validate prognostic accuracy of standardized radiological assessments of severe HIE on CT after CA. To our knowledge, no prospective studies have examined CTs acquired in CA patients at a pre-defined time-point ≥ 48 h post-arrest. The rationale behind the 48-hour time-point is based on retrospective studies demonstrating increased sensitivity of HIE for poor outcome prediction after the first 24 hours post-arrest.^11^, ^29^, ^31^, ^32^ The increasing sensitivity of late as compared to early (within the first hours after CA) head CT is in line with the pathophysiology of post-cardiac arrest brain injury, frequently developing over the first days post-arrest.^1^, ^33^ This is also demonstrated in studies using diffusion weighted imaging sequences in patients repeatedly examined with MRI and in decreasing GWR in patients repeatedly examined with CT.^31^, ^34^

Qualitative evaluation of HIE by local radiologists demonstrated a very high specificity in the TTM-trial, but since results were available to treating physicians, the risk of a self-fulfilling prophecy influencing outcome could not be excluded.^29^ In a recent guideline validation study from South-Korea where therapy is rarely withdrawn, Youn *et al.* reported a specificity of only 86% for the finding of a “poor CT” when evaluated by radiologists blinded to clinical data.^6^ Similar results have been reported for diffusion-weighted sequences on MRI.^35^ Although combinations of imaging techniques with other prognostic methods predicted poor outcome without false positive predictions in validation studies, the lack of a standardized radiological assessment of HIE may pose a risk for patients.^6^, ^29^, ^36^, ^37^ For this reason, since decisions on WLST within the TTM2 trial could also be based on CT findings, we will evaluate how well our radiological assessments correlate with blood levels of a surrogate marker of brain injury, the serum concentration of NFL. We have previously demonstrated that low levels of NFL predicted good outcome in 95% of patients, and that by combining biomarkers with other prognostic methods, the risk of false pathological CT evaluations could be minimized.^18^

Guidelines suggest “using the presence of a marked reduction of the GWR on brain CT within 72 h after ROSC in combination with other predictors for prognosticating a poor neurologic outcome in patients who are comatose after cardiac arrest and who are treated with TTM” (very low level of evidence).^2^ GWR cutoffs between 1.1 and 1.2 have demonstrated 100% specificity for poor outcome prediction.^2^ Our pilot study demonstrates that the GWR cutoff for 0% FPR varies with the anatomical localization of ROIs included in the measurement and is subject to interrater variability.^15^ Further, a quantitative assessment is rarely part of clinical routine and its implementation would require time-efficient tools that are easy to use. Previously, the 16 ROI model demonstrated lower levels of interrater variability compared to other models. Nonetheless, this method is not very well suited for practical use due to the high number of ROIs. The 8 ROI model only measuring densities at the basal ganglia level (BG) demonstrated similar prognostic accuracies and interrater variability as the 16 ROI model.^12^ We also include a simplified 4 ROI version at the basal ganglia level, which is the preferred GWR model by the examiners using GWR in clinical practice.^13^ However, since the simplified version demonstrated larger interrater variability in our pilot study, we want to validate whether this GWR model is indeed as accurate as the 8 BG model.

We previously found that a GWR below 1.1 predicted poor outcome with 100% specificity whilst a GWR below 1.15 in combination with severe HIE on the qualitative evaluation increased sensitivity and maintained this high specificity.^15^ Since a radiological evaluation always includes the qualitative assessment, GWR measurements should be used as an add-on for the highest accuracy.

Based on previous results, we include technical prerequisites for evaluation. Only CTs with axial slices of 4–5 mm and a tube voltage of 120kvP will be used in this study since HU are highly dependent on these parameters. We will also investigate to which extent the type of CT scanner used or whether residual contrast agent from coronary angiography may influence the reliability of our SOP.

### Strengths and limitations

Strengths include the prospective design and the blinded assessment by several examiners. As serum NFL samples were analysed after trial completion, we can compare CT evaluation with an independent marker of HIE severity which was not available during clinical decision-making.^24^ The TTM2 trial had a standardized and conservative approach to neurological prognostication with strict criteria for WLST.^16^ Evaluation of functional outcome was standardized and performed by experienced investigators blinded to clinical data.^16^, ^22^ The standardized criteria for radiological assessments presented in this manuscript were defined by radiologists and neurologists with clinical experience in radiological neuroprognostication and are based on results from pilot studies.^15^ We acknowledge, that these criteria partly reflect on our own clinical traditions, yet they are a step towards a standardization of head CT analysis for neuroprognostication.

Our study has several limitations: the TTM2 trial included adult patients with an out-of-hospital CA with a presumed cardiac origin, or with an unknown cause with a stable return to spontaneous circulation. Our results should be validated in other patient cohorts since results may vary. The results from local radiological evaluations were included when making decisions on level-of-care and the risk of a self-fulfilling prophecy cannot be excluded. We will therefore examine whether WLST for neurological reasons was performed in patients diagnosed with severe HIE on CT despite low serum levels of NFL as a surrogate marker of brain injury.

## Conclusion

The results from this prospective trial will provide a unique opportunity to validate a standardized approach to quantitative and qualitative radiological evaluations of HIE on CT for prediction of functional outcome in comatose cardiac arrest patients. We believe that our study will deliver clinically important information on an area where level of evidence is sparse.

## Funding

The TTM2 trial is supported by independent research grants from nonprofit or governmental agencies (the Swedish Research Council [Vetenskapsrådet], Swedish Heart–Lung Foundation, Stig and Ragna Gorthon Foundation, Knutsson Foundation, Laerdal Foundation, Hans-Gabriel and Alice Trolle-Wachtmeister Foundation for Medical Research, and Regional Research Support in Region Skåne) and by governmental funding of clinical research within the Swedish National Health Service. In addition, the CT substudy is supported by grants from the Bundy Academy, the Segerfalk Foundation and the Elsa Schmitz Foundation. MK is supported by the Laerdal Foundation and the Berlin Institute of Health Junior Digital Clinician Scientist Program. The authors are solely responsible for the design and conduct of this study, all study analyses, the drafting and editing of the manuscript, and its final contents.

## Conflicts of interest

CL declares institutional fees for lectures from Bard and Zoll. ND reports past lecture and travel fees for Bard and Zoll companies outside the present work. No other conflicts of interest were reported.
