# Supplementary material for: Prognostic accuracy of head computed tomography for prediction of functional outcome after out-of-hospital cardiac arrest: Rationale and design of the prospective TTM2-CT-substudy
Source: Resusc Plus. 2022 Oct 12;12:100316. doi: 10.1016/j.resplu.2022.100316 (PMC9576971; doi:10.1016/j.resplu.2022.100316)
Supplement: Supplementary data 1 [file mmc1.docx]

Supplementary content

**Prognostic accuracy of head computed tomography for prediction of functional outcome after out-of-hospital cardiac arrest**

**-Rationale and design of the prospective TTM2-CT-substudy**

Margareta Lang,^1^ Christoph Leithner,^2^ Michael Scheel,^3^ Martin Kenda^2,4^ Tobias Cronberg,^5^ Joachim During,^6^ Christian Rylander,^7^ Martin Annborn,^8^ Josef Dankiewicz,^9^ Nicolas Deye,^10^ Thomas Halliday,^11^ Jean-Baptiste Lascarrou,^12^ Thomas Matthew,^13^ Peter McGuigan,^14^ Matt Morgan,^15-17^ Matthew Thomas,^18^ Susann Ullén,^19^ Johan Undén,^20-21^ Niklas Nielsen,^8^ and Marion Moseby-Knappe.^5^

^1^Department of Clinical Sciences Lund, Radiology, Lund University, Helsingborg Hospital, Helsingborg, Sweden.

^2^Department of Neurology and Experimental Neurology, Charité-Universitätsmedizin Berlin, Germany.

^3^Department of Neuroradiology, Charité-Universitätsmedizin Berlin, Germany.

^4^Berlin Institute of Health at Charité, Universitätsmedizin Berlin, Germany.

^5^Department of Clinical Sciences Lund, Neurology, Lund University, Skåne University Hospital, Lund, Sweden.

^6^Department of Clinical Sciences Lund, Anaesthesia and Intensive Care, Lund University, Skåne University Hospital, Malmö, Sweden.

^7^Department of Surgical sciences, Anaesthesia and intensive care, Uppsala University, Uppsala, Sweden.
^8^Department of Clinical Sciences Lund, Anesthesia & Intensive care, Lund University, Helsingborg Hospital, Helsingborg, Sweden

^9^Department of Clinical Sciences Lund, Cardiology, Lund University, Skåne University, Lund, Sweden.

^10^Department of Medical and Toxicological Intensive Care Unit, Lariboisière Hospital, Paris, France.
^11^Department of Operation and Intensive Care, Linköping University Hospital, Linköping, Sweden.

^12^Médecine Intensive Réanimation, University Hospital Center, Nantes, France.

^13^Intensive Care Unit, University Hospitals, Bristol and Weston, England, United Kingdom.

^14^Regional Intensive Care Unit, Royal Victoria Hopsital, Belfast, Northern Ireland, United Kingdom.

^15^Department of Intensive Care, the Royal Perth Hospital, Perth, Australia.

^16^Department of Intensive Care, the University Hospital of Wales, Cardiff, United Kingdom.

^17^School of Medicine, Curtin University, Perth, Australia.

^18^University Hospitals, Bristol and Weston, United Kingdom.

^19^Clinical Studies Sweden ‑ Forum South, Skåne University Hospital, Lund, Sweden.

^20^Department of Clinical Science Lund, Lund, Sweden.

^21^Department of Operation and Intensive Care, Hallands Hospital Halmstad, Halmstad, Sweden.

Corresponding author:

Margareta Lang, MD
Helsingborg Hospital
Department of Radiology,
252 23 Helsingborg, Sweden
Phone.:+46-42-4062826
Fax. +46-42-4061208
E-mail: margareta.lang@med.lu.se

**S1. SOP qualitative CT analysis**

**Prerequisites for qualitative analysis**

1. Technical details:
   1. Head CT including the entire brain
   2. 4-5mm axial slice reconstruction
   3. 120 KvP (Peak kilovoltage)
2. No imaging artifacts precluding analysis, e.g. motion artifacts, radiodense foreign material.
3. No brain diseases other than HIE interfering with analysis, for example any significant acute or chronic brain disease precluding analysis such as intracranial haemorrhage, stroke, tumor, extensive calcification. Importantly, CTs with moderate atrophy, moderate vascular leukoencephalopathy, brain atrophy or chronic strokes not affecting analysis of grey-white matter distinction or sulcal effacement at the basal ganglia level and bilateral frontoparietal regions may be used for analysis.


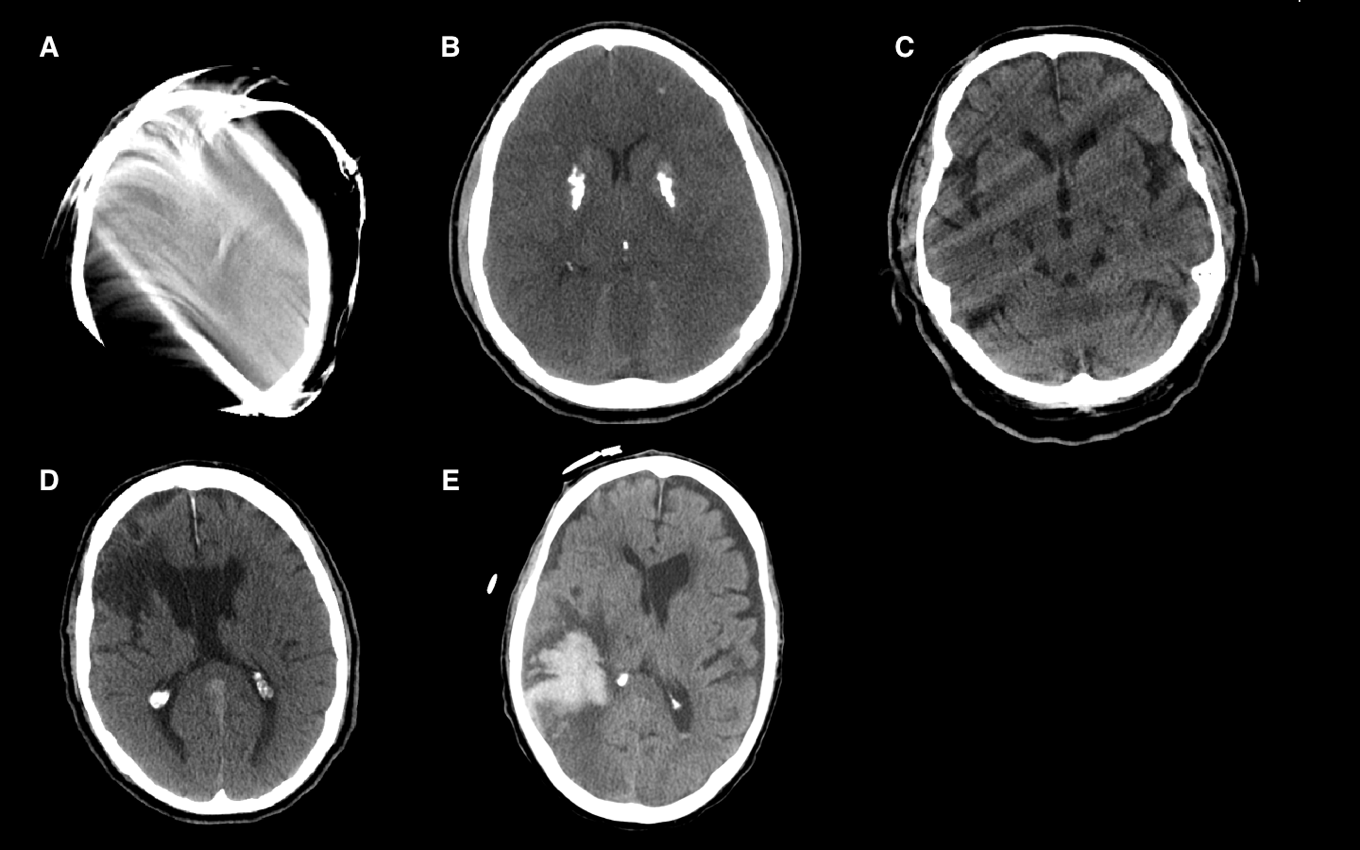


Figure 1 – Example Images for Exclusion (A) Distorted image, (B) calcification of the basal ganglia, (C) Severe motion artifacts, (D) Large old ischaemic lesion, (E) Intracerebral haemorrhage, (modified from Kenda et al, 2021)

**Qualitative head CT Analysis**

Image evaluation should start using a standard brain windowing and then adapt to optimize visibility of grey-white matter differentiation.

The entire brain should be evaluated following the checklist below. The focus should be on distinction of grey and white matter and on supratentorial sulcal effacement. These characteristics should be evaluated at 4 levels (see checklist below): 1) brainstem + cerebellum, 2) basal ganglia, 3) cortex at corona radiata, 4) high convexity cortex.

The main question to be answered after evaluation of the entire head CT is ‘Are there definite signs of severe HIE’? This question should be answered with ‘yes’, if complete or near-complete loss of grey-white distinction is noted both (!) in 1) the basal ganglia and 2) in the frontoparietal lobes bilaterally with additional signs of brain swelling/sulcal effacement. Severe HIE can also be diagnosed with complete sulcal effacement when residual grey-white distinction is present. Consider patient age for assessment of age-specific brain volume. Caution should be taken not to diagnose severe HIE in young patients with physiologically high brain volume, small ventricles, only few visible sulci and normal grey-white matter distinction.

Additional characteristics of severe HIE may include ‘Pseudo-SAH’ (hyperdense CSF signal in basal cisterns), ‘reversal sign’ (lower radiodensity of basal ganglia grey matter structures as compared to white matter structures), ‘white cerebellum sign’ (higher radiodensity of cerebellum as compared to cerebral hemispheres) and contrast enhancement of grey matter structures, e.g. after coronary angiography.


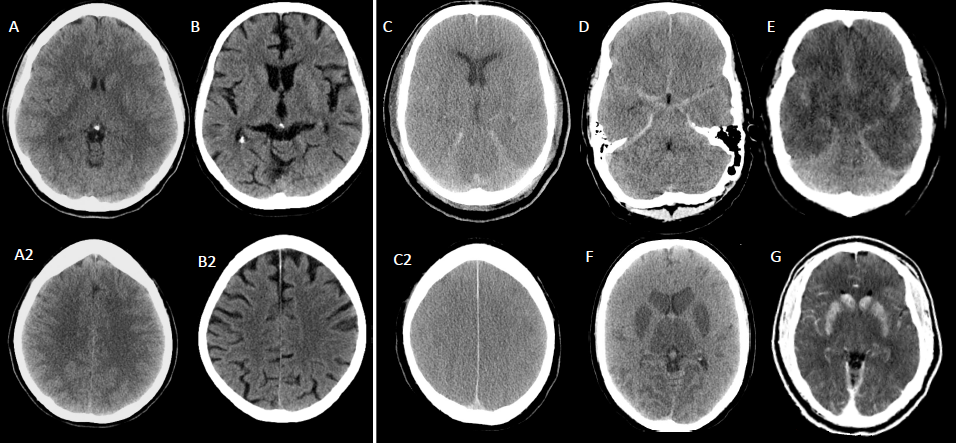


Figure 2 – Example Images. A/A2 – young patient with physiologically small CSF spaces and intact grey-white distinction; B/B2 – older patient with some brain atrophy and intact grey-white distinction; C/C2 loss of grey-white distinction and complete sulcal effacement, D – Pseudo-SAH, E- white cerebellum sign, F – reversal sign with hypodensity of basal ganglia grey matter structures, G – contrast enhancement of basal ganglia grey matter structures after coronary.

**Checklist - SOP for qualitative analysis in the TTM2 CT-Substudy**

Patient: _____________ Rater: ____________

**
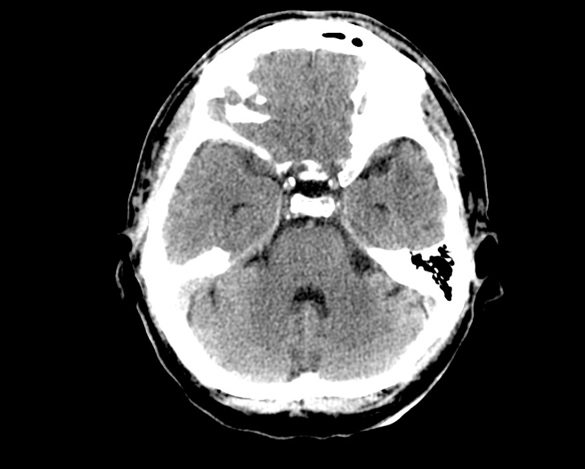
Prerequisites CT Levels**

Artifacts precluding analysis  yes no

Brain diseases precluding analysis  yes no

Residual contrast agent visible  yes no

_ _ _ _ _ __ _ __ _ __ _ __ _ __ _ __ _ __ _ __ _ __ _ __ _ __ _ __ _ _

**Qualitative Analysis**

Start using standard brain window and then adapt to optimize visibility of grey-white matter differentiation. *Evaluate axial images at these 4 different levels. Consider the best grey-white-differentiation, best visibility of sulci*


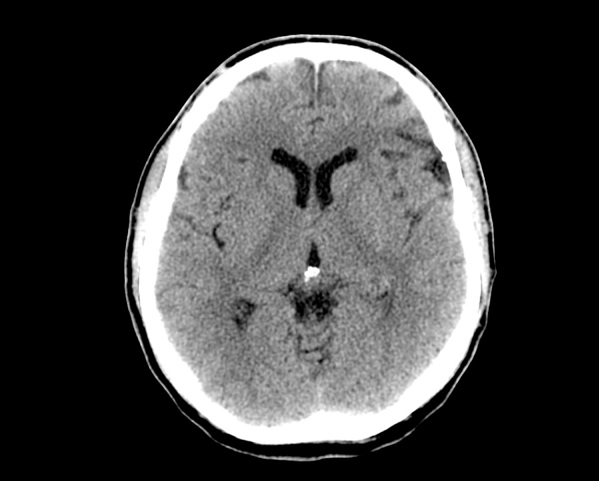


1 - Brain stem + Cerebellum

Effacement of CSF spaces  yes no

Pseudo-SAH  yes no

White Cerebellum Sign  yes no

2 - Basal ganglia

Bilateral loss of grey-white distinction  yes no

Bilateral sulcal effacement  yes no

Reversal sign  yes no


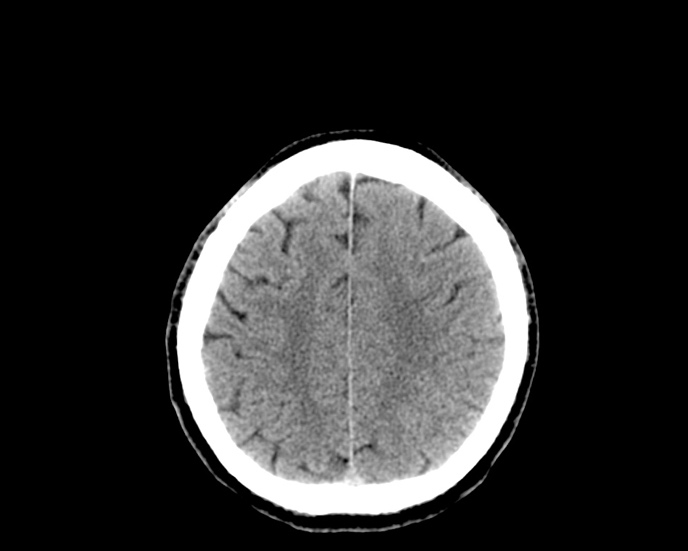
3 - Frontoparietal cortex corona radiata level

Bilateral loss of grey-white distinction  yes no

Bilateral sulcal effacement  yes no

4 - High convexity cortex

Bilateral loss of grey-white distinction  yes no

Bilateral sulcal effacement  yes no

_ _ _ _ _ __ _ __ _ __ _ __ _ __ _ __ _ __ _ __ _ __ _ __ _ __ _ __ _ _

**Considering all 4 levels**

Complete loss of grey-white distinction  yes no


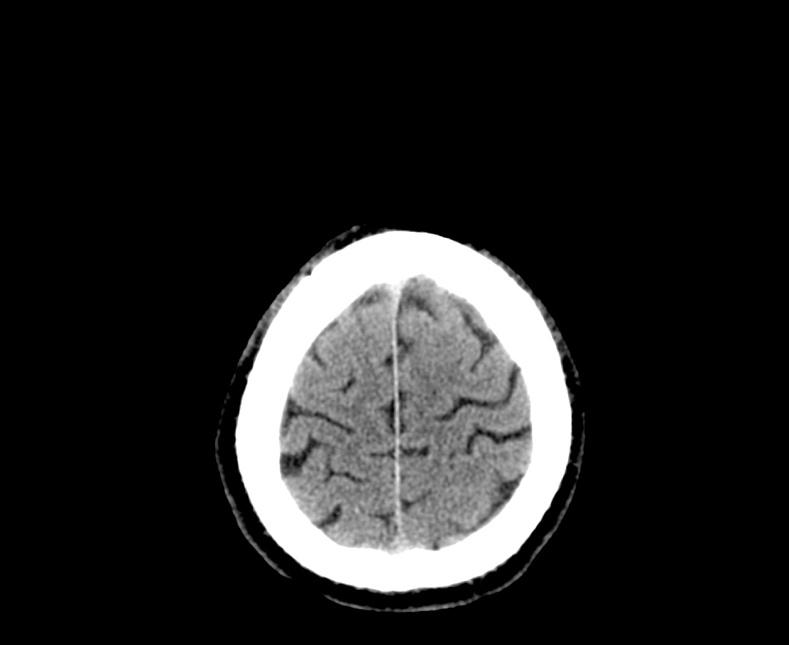
Complete effacement of all sulci  yes no

**Result of qualitative analysis**

Definite severe HIE: *complete or near complete loss of grey-white distinction in the basal ganglia and in the frontoparietal cortex with additional evidence of brain swelling/sulcal effacement. Consider patient age while evaluating.*

- Definite signs of severe HIE
- No definite signs of severe HIE

**S2. SOP quantitative CT analysis**

**Grey-white matter ratio (GWR) determination**

Before starting GWR determination, perform the qualitative evaluation and enter the results into the electronic case report form.

GWR determination is carried out by manual placement of 8 circular ROIs with an area of 0.1cm^2^.

Hounsfield Units should be displayed to ensure ROI placement in an area where radiodensity is representative of that target brain region. Focal hypo- or hyperdensities, e.g. resulting from small vascular lesions, calcifications or noise must be avoided.

**
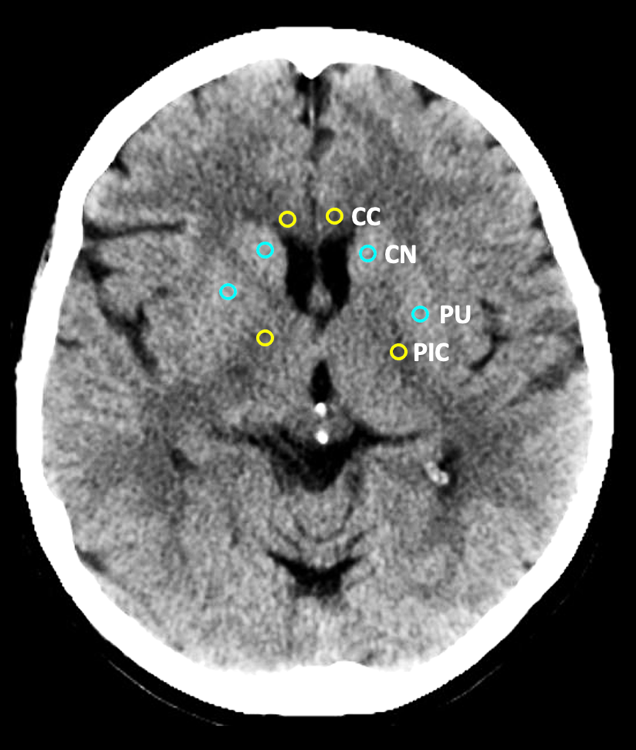
**

Figure 1 – ROI Placement for GWR determination at the basal ganglia level. Blue – grey matter ROIs, yellow – white matter ROIs. CC; genu of the corpus callosum, CN; head of the caudate nucleus, PU; putamen, and PIC; posterior limb of the internal capsule.

**8 ROI basal ganglia GWR (8 BG)**

All axial slices containing basal ganglia structures should be evaluated and ROIs placed bilaterally in the slice best representative of that target region. Thus, these 8 ROIs may be placed in different slices:

- Putamen
- Posterior limb of the internal capsule
- Head of the caudate nucleus
- Genu of the corpus callosum

In case of complete loss of grey-white distinction, use ventricles and midline as landmarks. In some patients with severe HIE radiodensity is similar in grey and white matter, exact location of target regions cannot always be determined. Nonetheless, ROIs should be placed and patients should not be excluded from GWR determination. The GWR of the 8 BG model will be calculated with the radiodensities measured at the ROI as follows:

$$8 ROI BG GWR= \frac{{CN}_{right}+{CN}_{left}+ {PU}_{right}+ {PU}_{left}}{{CC}_{right}+ {CC}_{left}+ {PIC}_{right}+ {PIC}_{left}}$$
